# The Role of G-proteins and G-protein Regulating Proteins in Depressive Disorders

**DOI:** 10.3389/fphar.2018.01289

**Published:** 2018-11-13

**Authors:** Nicolas B. Senese, Mark M. Rasenick, John R. Traynor

**Affiliations:** ^1^Department of Physiology and Biophysics, University of Illinois at Chicago, Chicago, IL, United States; ^2^Jesse Brown VA Medical Center, Chicago, IL, United States; ^3^Department of Pharmacology and Edward F. Domino Research Center, University of Michigan, Ann Arbor, MI, United States; ^4^Department of Psychiatry, University of Illinois at Chicago, Chicago, IL, United States

**Keywords:** G-protein, RGS, antidepressant, depression, GPCR

## Abstract

Progress toward new antidepressant therapies has been relatively slow over the past few decades, with the result that individuals suffering from depression often struggle to find an effective treatment – a process often requiring months. Furthermore, the neural factors that contribute to depression remain poorly understood, and there are many open questions regarding the mechanism of action of existing antidepressants. A better understanding of the molecular processes that underlie depression and contribute to antidepressant efficacy is therefore badly needed. In this review we highlight research investigating the role of G-proteins and the regulators of G-protein signaling (RGS) proteins, two protein families that are intimately involved in both the genesis of depressive states and the action of antidepressant drugs. Many antidepressants are known to indirectly affect the function of these proteins. Conversely, dysfunction of the G-protein and RGS systems can affect antidepressant efficacy. However, a great deal remains unknown about how these proteins interact with antidepressants. Findings pertinent to each individual G-protein and RGS protein are summarized from *in vitro*, *in vivo*, and clinical studies.

## Introduction

Major depressive disorder (MDD) is one of the most prevalent psychiatric disorders with over 16% of adults in the US experiencing a depressive event within their lifetime, and over half of these events leading to severe or very severe role impairment (Kessler et al., 2003; García-Velázquez et al., 2017). While a multitude of antidepressant drugs are now available, no one treatment is fully effective in all patients, with about one third failing to remit even after 4th line treatments (Insel and Wang, 2009). This high rate of treatment failure combined with the high prevalence of depressive disorders highlights the need not only for improved treatment options, but also for a better understanding of the molecular and cellular factors that determine whether a given treatment will succeed or fail.

In this review we highlight the role of G-proteins and their signaling partners, especially the Regulators of G-protein Signaling (RGS) proteins, in both the etiology and treatment of depression.

## GPCRS in Depression

The vast majority of drugs prescribed for depressive disorders either interact directly with G-protein coupled receptors (GPCRs), e.g., buspirone with the 5-HT1A receptor or aripiprazole with a multitude of monoaminergic GPCRs, or indirectly regulate GPCR function by affecting endogenous neurotransmitter levels, e.g., selective serotonin reuptake inhibitors (SSRIs) such as fluoxetine and monoamine oxidase inhibitors (MAOIs) such as selegiline (Kantor et al., 2015).

**FIGURE 1:**
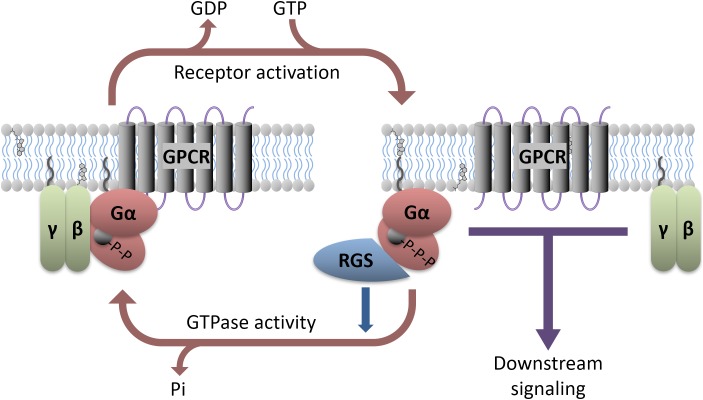
Heterotrimeric G-protein signaling with RGS regulation. GPCR activation, either due to agonist binding or constitutive activity, causes downstream signaling through both the α and βγ subunits. Various antidepressants modulate this process directly (e.g., Buspirone) or indirectly (e.g., SSRIs). RGS proteins interact with active Gα and accelerate its GTPase activity, facilitating a return to the GDP-bound inactive state. Preclinical models suggest that direct manipulation of the RGS or G-proteins can affect antidepressant response.

G-protein coupled receptors are 7-transmembrane domain proteins that form a multi-protein complex with members of an intracellular family of heterotrimeric G-proteins comprised of a Gα subunit and a βγ dimer (Figure 1). There are several members of the Gα family that couple to different cohorts of effectors in the cell. These include the so-called inhibitory Gα proteins (Gα_i/o_ and Gα_z_), which inhibit adenylyl cyclase to decrease cyclic adenosine monophosphate (cAMP), the adenylyl cyclase stimulatory Gα_s_ and Gα_olf_, Gα_q_ which activates phospholipase C, and Gα_12/13_ that couple to the Rho family of small GTPases. There are 5 β subunits and 11 γ subunits that can be engaged to make the associated βγ dimer in mammals. There is selectivity of receptors for particular types of Gα proteins, and also for activation or inhibition of downstream effectors in the cell. Following GPCR activation by endogenous neurotransmitters or exogenous agonists both the Gα subunit and the Gβγ complex functionally dissociate from the receptor and go on to stimulate or inhibit a range of intracellular effectors. The Gα activation process involves a loss of bound GDP (inactive form) in exchange for GTP (active form). Signaling is terminated by the hydrolysis of the bound GTP back to GDP by the intrinsic GTPase activity of the Gα subunit. However, for certain Gα proteins this enzymatic process is slow. To accelerate this process a regulator of G-protein signaling (RGS) protein binds to the active GTP-bound Gα and facilitates its inactivation, acting as a GAP (GTPase accelerating protein; Figure 1). This allows return to the resting, inactive state. The inactive GDP-bound Gα subunit can then recouple with both the Gβγ complex and receptor until the receptor is again activated and the cycle repeats. Although RGS proteins can substantially limit Gα_i/o_ and Gα_q_ signaling (see Oldham and Hamm, 2008 for review) their impact on Gα_s_ mediated signaling is less pronounced. While an RGS for Gα_s_ has been identified, it is not clear that this protein facilitates the GTPase activity of Gα_s_ (Zheng et al., 2001; Ha et al., 2015).

In addition to mediating the actions of antidepressant drugs, many GPCRs have been associated with the development of depression. Aberrations in both α- and β-adrenergic receptor signaling have been found in depressed patients (Matussek et al., 1980; Ebstein et al., 1988) and the brains of suicide victims consistently have alterations in 5-HT1A receptor expression (and various nuclear receptors) resembling the alterations produced by chronic stress in animal models (López et al., 1998). In contrast, study of 5-HT2A and 5-HT2C receptor expression levels has produced inconsistent results (see Stockmeier, 2003 for review). Nonetheless, a 5-HT2C receptor polymorphism in the N-terminal extracellular domain has been associated with MDD in a large population study (Lerer et al., 2001). Preclinical models also implicate the 5-HT1B receptor both in the genesis of depressive states and in antidepressant action (Svenningsson et al., 2006). Polymorphisms in both the dopamine D3 and D4 receptors have been correlated with the development of MDD (Dikeos et al., 1999; López León et al., 2005), while D1 and D2 receptors have instead been linked to bipolar disorder (Massat et al., 2002; Dmitrzak-Weglarz et al., 2006). Interestingly the GABA-B receptor agonist baclofen produces a transient depressive state in some patients (Post et al., 1991), suggesting this receptor may play a role in MDD. A corticotropin-releasing hormone receptor 1 antagonist has also been found to have antidepressant activity (Zobel et al., 2000), in agreement with predictions from preclinical studies (Mansbach et al., 1997).

Downstream signaling bias is also affected by proteins in complex with the receptor. For example, the 5-HT1A receptor can form receptor/receptor complexes with the 5-HT7 receptor, the ghrelin receptor family member GPR39, or fibroblast growth factor receptor 1 (FGFR1), with each heterodimer complex producing a unique downstream signaling profile (Renner et al., 2012; Tena-Campos et al., 2015; Borroto-Escuela et al., 2017). The 5-HT1A/5-HT7 heterodimer complex promotes internalization of the 5-HT1A receptor (Renner et al., 2012), suggesting that the high degree of colocalization between these receptors in the dorsal raphe nucleus may contribute to 5-HT1A autoreceptor desensitization observed during SSRI treatment (Naumenko et al., 2014). These results imply that strategies which promote 5-HT1A/5-HT7 heterodimer formation could facilitate the antidepressant effects of SSRIs by attenuating 5-HT1A autoreceptor activity. In contrast, combined 5-HT1A receptor and FGFR1 agonist treatment fails to produce antidepressant-like behavior in rats with low hippocampal 5-HT1A/FGFR1 heterodimer expression (Borroto-Escuela et al., 2017), while these agonists produce robust antidepressant-like effects in rats with increased dimer formation. These heterodimer dependent behavioral effects are likely mediated by a reduced ability of the 5-HT1A/FRGR1 heterodimer to activate G-protein coupled inwardly rectifying potassium (GIRK) channels, compared to the free 5-HT1A receptor (Borroto-Escuela et al., 2017). These results suggest that 5-HT1A receptor stimulated GIRK activity in the hippocampus is in fact detrimental to antidepressant activity, and that agonists which preferentially couple to 5-HT1A/FGFR1 heterodimers would be superior to unbiased ligands. For a recent review on how GPCR oligomerization can affect receptor activity, see Szafran et al. (2013) and Farran (2017) for review of GPCR heterodimers in depression.

The above discussion suggests that insight into the roles of specific G-proteins and their cognate RGS proteins in antidepressant action may facilitate future antidepressant drug development. Furthermore, studying dysfunction of these systems in the depressed brain may provide insight into the etiology of depression.

## G-Protein Subunits in Depression and Antidepressant Action

### G-protein Expression Levels

In preclinical studies, central nervous system (CNS) G-protein expression levels do not appear to change consistently as a result of antidepressant drug treatment. Gα_s_, Gα_o_ and Gα_i_ mRNA expression in the rat hippocampus remain constant following chronic treatment with the tricyclic antidepressant imipramine (Lasoń and Przewłocki, 1993). However, levels of G-protein mRNA and protein expression do not always have a strong correlation (Krumins and Gilman, 2006), and so these results do not necessarily reflect the amount of G-protein present. Chronic treatment with the dual serotonin norepinephrine reuptake inhibitor (SNRI) amitriptyline, the tricyclic antidepressant desipramine, the MAOI tranylcypromine or electroconvulsive shock did not affect protein levels of Gα_s_, Gα_o_, Gα_i_ or Gβ in the rat cerebral cortex (Chen and Rasenick, 1995a; Emamghoreishi et al., 1996; Dwivedi and Pandey, 1997). In contrast, brief treatment with the MAOI antidepressant phenelzine increased Gα_i2_ protein expression in the rat cortex and hippocampus without affecting Gα_s_, Gα_o_, Gα_q_ or Gα_i1_ expression in any brain region (Dwivedi and Pandey, 1997). However, this does not appear to be a conserved effect for all MAOI antidepressants on Gα_i2_, as chronic treatment with tranylcypromine did not affect cortical Gα_i2_ expression while chronic clorgiline instead produced a small decrease (Lesch et al., 1991; Emamghoreishi et al., 1996). Three-week treatment with various tricyclic antidepressants (imipramine, desipramine, or chlomipramine) produced slight increases of brain Gα_o_ and decreases of Gα_s_ and Gα_i_, although the magnitude of these changes (∼10–30% from baseline) may not be great enough to produce functional consequences (Lesch et al., 1991). Note that it has been suggested that Gα_s_ expression is over 30-fold higher than expression of downstream effectors (Ostrom et al., 2000), thus minor variations in the expression of Gα_s_ are not expected to influence downstream signaling. Furthermore, tricyclics such as desipramine and amitriptyline had no effect on G-protein expression (Chen and Rasenick, 1995a; Emamghoreishi et al., 1996).

On the other hand, a series of post-mortem studies examining the involvement of G-proteins in depressive states contrast with the findings from pre-clinical studies discussed above. Post-mortem studies indicate that a downregulation of Gα_o_ and Gα_i2_ protein and mRNA co-occurs with an upregulation of Gα_s_ protein levels and mRNA in the prefrontal cortex of adult suicide cases (Dwivedi et al., 2002). This fits with data from Gα_i2_ knock-out mice which show that loss of Gα_i2_ contributes to depressive behaviors (Talbot et al., 2010), suggesting that the observed alterations of Gα_i2_ in these subjects may have contributed to their pathology. However, the work of Donati et al. (2008) shows only minor changes in Gα_s_ expression and none in other Gα proteins. Thus, there is no consistent effect on G-protein expression in the brain following chronic antidepressant treatment, the effects that have been seen are not consistent between antidepressant drugs with similar pharmacology, and any changes observed are of relatively small magnitude.

### Effects on Gα_s_ Localization and Signaling

Despite the lack of any clear effect on G-protein expression levels, chronic but not acute antidepressant drug treatment (including amitriptyline, desipramine and iprindole) increases cAMP concentrations in a Gα_s_ dependent manner in the rat brain, but not liver or kidney (Menkes et al., 1983; Ozawa and Rasenick, 1989; De Montis et al., 1990). In addition to antidepressant drug treatments, chronic electroconvulsive treatment increases coupling between Gα_s_ and adenylyl cyclase (Ozawa and Rasenick, 1991). Consistent with this increased adenylyl cyclase activity, increased activity of cAMP dependent kinases (e.g., Protein kinase A) have also been observed in the rat brain following chronic antidepressant treatment. These changes occurred with chronic but not acute treatment with desmethylimipramine, and were seen in the cerebral cortex but not hippocampus, striatum or cerebellum (Perez et al., 1989). This suggests a more general role for brain Gα_s_/adenylyl cyclase coupling in antidepressant action downstream of their better characterized direct effects on transporters and GPCRs.

**FIGURE 2:**
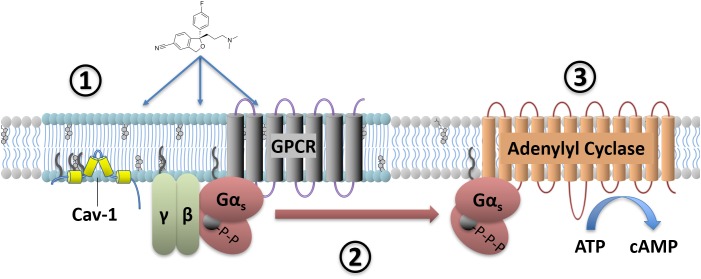
Antidepressant effects on Gα_s_ signaling. Antidepressant drugs (e.g., escitalopram, depicted) slowly accumulate in lipid raft domains **(1)**. These areas are defined in part by high cholesterol content, detergent insolubility, and expression of membrane proteins such as Caveolin-1. Antidepressant accumulation causes a translocation of Gα_s_ from lipid raft to non-raft membrane **(2)**, where coupling to downstream effectors such as adenylyl cyclase increases. This facilitates downstream signaling, including increased cAMP production **(3)**.

In order to understand how antidepressant drugs affect G-protein signaling, it is necessary to consider not only the expression levels of these proteins and their binding partners, but also their subcellular localization in microdomains. Within the plasma membrane bilayer there are lipid raft microdomains which contain an increased proportion of both cholesterol and sphingomyelin (Simons and Toomre, 2000) and the scaffolding protein Caveolin 1 (Figure 2). G-proteins are known to accumulate in these lipid raft domains, with Gα_s_, Gα_q,_ and Gα_i/o_ subunits all found at higher concentrations in these regions (Pesanová et al., 1999; Dunphy et al., 2001; Allen et al., 2005). These microdomains can affect G-protein mediated signaling, with either faciliatory or inhibitory effects on signaling depending on the G-protein. For example, localization to raft regions inhibits the ability of Gα_s_ proteins to increase cAMP levels through adenylyl cyclase activation (Allen et al., 2009), while raft localization of Gα_q_ greatly enhances signaling downstream of 5-HT2A receptor activation (Rybin et al., 2000; Bhatnagar et al., 2004; Allen et al., 2007 for review). In addition to an upregulation of Gα_s_ protein expression, a shift in Gα_s_ subcellular membrane localization also occurs in the brains of depressed humans. Thus, compared to non-psychiatric control subjects there was an approximately two-fold increase in the localization of Gα_s_ to lipid raft domains in both cerebral cortex and cerebellum from suicide cases with documented depression (Donati et al., 2008). As these lipid raft domains are known to inhibit signaling downstream of Gα_s_ (Rybin et al., 2000; Miura et al., 2001; Head et al., 2006; Allen et al., 2009), including coupling to adenylyl cyclase, this increased lipid raft localization likely indicates decreased Gα_s_ signaling in the depressed brain. In fact, a Gα_s_ dependent adenylyl cyclase dysfunction in the depressed brain is supported by impairment in the ability of forskolin to stimulate adenylyl cyclase activity in post-mortem tissue from individuals who completed suicide (Cowburn et al., 1994). This loss of adenylyl cyclase activity is associated with decreased expression and activity of the cAMP dependent kinase (PKA) in the frontal cortex, but not hippocampus of suicide completers (Pandey et al., 2005).

Preclinical and *in vitro* models suggest that antidepressants cause both increased association of Gα_s_ with adenylyl cyclase (Table 1 and Figure 2) and reduce the relative amount of Gα_s_ localized in lipid rafts (Chen and Rasenick, 1995a,b; Toki et al., 1999; Donati and Rasenick, 2005; Allen et al., 2009; Zhang and Rasenick, 2010; Singh et al., 2018; Wray et al., 2018). Both of these effects of antidepressants would in theory directly counteract changes detected in post-mortem brain tissue from suicide completers, namely impaired adenylyl cyclase activity and increased accumulation of Gα_s_ in lipid rafts (Cowburn et al., 1994; Donati et al., 2008). While these results predict that antidepressant treatment should correct these deficits observed in the depressed brain, this hypothesis has not yet been fully tested.

**Table 1:** Antidepressants effects on Gα_s_ signaling.

	Gα_s_ Translocation	Gα_s_ Membrane mobility	cAMP	Reference
**Antidepressants**				
Amitriptyline	+		+	Menkes et al., 1983; Ozawa and Rasenick, 1989; Chen and Rasenick, 1995a,b; Toki et al., 1999
Bupropion		+		Czysz et al., 2015
Desipramine	+	+	+	Menkes et al., 1983; Ozawa and Rasenick, 1989; Chen and Rasenick, 1995a,b; Toki et al., 1999; Donati and Rasenick, 2005; Czysz et al., 2015
Electroconvulsive Therapy			+	Menkes et al., 1983; Ozawa and Rasenick, 1991; Chen and Rasenick, 1995a
Escitalopram	+	+	+	Zhang and Rasenick, 2010; Czysz et al., 2015; Donati et al., 2015; Erb et al., 2016; Singh et al., 2018
Fluoxetine	+	+		Toki et al., 1999; Donati and Rasenick, 2005; Czysz et al., 2015
Imipramine	+	+	+	Menkes et al., 1983; Czysz et al., 2015; Singh et al., 2018
Iprindole	+		+	Menkes et al., 1983; Ozawa and Rasenick, 1989; Toki et al., 1999
Phenelzine		+		Czysz et al., 2015
Sertraline		+		Czysz et al., 2015
Tianeptine		+		Czysz et al., 2015
Venlafaxine		+		Czysz et al., 2015
**Putative antidepressants**				
ABT 200			+	Chen and Rasenick, 1995a
Ketamine	+	+	+	Wray et al., 2018
Tubastatin-A	+	+		Singh et al., 2018
**Non-antidepressants**				
Amphetamine		-	-	Chen and Rasenick, 1995a
Chlorpromazine	-			Toki et al., 1999
Diazepam		-		Czysz et al., 2015
Haloperidol		-		Czysz et al., 2015
Lithium	-	-		Donati et al., 2015
LY368514	-			Donati et al., 2015
Olanzapine		-		Czysz et al., 2015
R-citalopram	-	-	-	Zhang and Rasenick, 2010; Czysz et al., 2015
Valproate	-	-		Donati et al., 2015

*Many antidepressant drugs are known to affect Gα_*s*_ localization and signaling. These effects include a reduction of Gα_*s*_ localized to lipid raft membrane regions, alongside increased Gα_*s*_ found in non-raft membranes (**Gα_*s*_ Translocation** column), reduced Gα_*s*_ lateral membrane mobility following antidepressant treatment, quantified by Fluorescence Recovery After Photobleaching (Gα_*s*_ membrane mobility column), and a faciliatory effect on cAMP accumulation (**cAMP** column). In general, these effects have been observed with antidepressant drugs, but not closely related analogues. For example, the antidepressant Escitalopram is known to produce these effects, while the inactive stereoisomer, R-citalopram, does not. A number of compounds with putative antidepressant qualities, including Tubastatin-A, ABT 200, and ketamine also show this Gα_*s*_ mediated antidepressant biosignature, suggesting these readouts may be useful for detecting novel antidepressants. A ’+’ in each column indicates this drug is known to cause this effect, while a ’-’ indicates this drug does not. Blank entries indicate that data has not been published for this effect.*

It is noteworthy, however, that recent PET imaging studies reveal decreased [^11^C]-Rolipram (a phosphodiesterase-4 inhibitor) binding in all brain regions of depressed subjects (Fujita et al., 2017). Subsequent to successful antidepressant treatment [^11^C]-Rolipram binding returns to baseline throughout the CNS (Fujita et al., 2017). This is consistent with the predictions from the work described above, as [^11^C]-Rolipram binding is known to reflect levels of cAMP (Hoffmann et al., 1998), due to a feedback mechanism involving cAMP, PKA, and phosphodiesterase-4 (Houslay et al., 1998).

In cellular models, multiple G-protein subtypes accumulate in lipid rafts and antidepressant drug treatment reduces the amount of lipid raft associated Gα_s_ without changing the abundance of other G-proteins in the rafts (Toki et al., 1999; Donati and Rasenick, 2005). This reduction in lipid raft Gα_s_ content occurs without changes in the overall expression level of Gα_s_ protein, or in the expression of other G-proteins including Gα_i_, Gα_o_ or Gβ (Chen and Rasenick, 1995b). The shift of Gα_s_ from lipid raft regions to non-lipid raft regions coincides with increased coupling between Gα_s_ and adenylyl cyclase as well as increased adenylyl cyclase activation and cAMP accumulation (Table 1; Chen and Rasenick, 1995a,b). Indeed, antidepressants including the tricyclic desipramine (Czysz et al., 2015), the SSRI escitalopram (Erb et al., 2016), and the NMDA antagonist ketamine (Wray et al., 2018) reduce the lateral membrane mobility of Gα_s_ (Table 1). This likely reflects increased association with the large, relatively immobile adenylyl cyclase protein following Gα_s_ translocation out of lipid raft domains (Czysz et al., 2015). These effects occur with tricyclic antidepressants, SSRIs and atypical antidepressants, suggesting a potential conserved antidepressant mechanism independent of the known sites of action.

Importantly, the transfer of Gα_s_ out of lipid raft domains occurs following chronic treatment with the antidepressant (S)-stereoisomer of the SSRI citalopram, but not the (R)-stereoisomer which lacks antidepressant effects (Zhang and Rasenick, 2010). This stereospecific effect of citalopram occurs in C6 cells lacking the serotonin transporter, suggesting that Gα_s_ translocation out of lipid rafts occurs due to interaction of the antidepressant with some other protein target. Furthermore, antidepressant drugs with diverse mechanisms of action (including desipramine, reboxetine and fluoxetine) themselves accumulate in these lipid raft domains over time (Eisensamer et al., 2005). Initially these drugs distribute evenly throughout the plasma membrane, but gradually partition into lipid rafts over a 3-day treatment period (Erb et al., 2016). While together these data suggest that a specific binding site for antidepressants within lipid rafts may exist, a suitable candidate site has yet to be identified.

Therefore, in general antidepressant drugs liberate Gα_s_ from the inhibitory effects of lipid raft localization (Head et al., 2006) allowing this subunit to signal more effectively through downstream effectors including adenylyl cyclase (Table 1 and Figure 2). These effects are relatively specific to the brain as peripheral tissues do not show the same response, and even within the brain there is regional specificity as brain regions other than the cerebral cortex show reduced Gα_s_ translocation, if any (Dwivedi et al., 2002). This provides a plausible mechanism for the long-recognized ability of antidepressant drugs to increase the coupling between Gα_s_ and adenylyl cyclase (Menkes et al., 1983). This enhancement occurs only following extended antidepressant treatment, consistent with the hysteresis observed between the initiation of antidepressant treatment and the onset of therapeutic effects (Chen and Rasenick, 1995a). Notably, ketamine, which is reported to have a rapid therapeutic onset, shows the antidepressant biosignature described above after only 15 min of treatment (Wray et al., 2018).

Although Gα_s_ coupling to adenylyl cyclase is unaffected by antidepressant treatment in peripheral tissues (e.g., liver and kidney; Menkes et al., 1983), a variety of blood cells undergo changes similar to those observed in the brain. Lymphocytes and platelets from depressed subjects have diminished cAMP production due to decreased Gα_s_-adenylyl cyclase coupling (Hines et al., 2005) and this is resolved in subjects who respond to antidepressants (Pandey et al., 1985; Mooney et al., 2013). These data raise the possibility of using Gα_s_ signaling changes in peripheral blood cells to test for depression and antidepressant response, a strategy that has already seen some success with other markers detected in patient blood samples (Rivera-Baltanas et al., 2014).

Together the above discussion suggests a dysregulation of G-protein signaling in the brains of depressed individuals. The dysregulation likely involves changes in overall G-protein expression, as well as translocation of Gα_s_ into a more restrictive membrane microenvironment where signaling to downstream effectors is inhibited, with apparent consequences for cyclase dependent signaling pathways including protein kinase A. It remains unclear whether these changes contribute to suicidal behavior and depression, or are simply correlated with the expression of these states without a causative effect. In either case the existence of these changes may allow for new strategies to diagnose and potentially treat depressive disorders.

## RGS Proteins in Depression and Antidepressant Drug Action

As mentioned earlier RGS proteins are essential modulators of signaling downstream of the G-proteins discussed above. There are more than 20 members of the RGS protein family, designated by the presence of a 120 amino-acid RH domain and divided into subfamilies based on their structures outside of the RH domain (Hollinger and Hepler, 2002; Traynor and Neubig, 2005). This variety indicates that RGS proteins have some degree of specificity for which Gα subunits they can regulate. For example, RGS4 and RGS8 potently inhibit signaling downstream of Gα_i2_ *in vitro* while RGS7 has no effect (Talbot et al., 2009). RGS-PX1 and RGS-PX2 regulate signaling downstream of Gα_s_ (Zheng et al., 2001; Ha et al., 2015), while RGS2 preferentially modulates Gα_q_ signaling (Heximer et al., 1997). RGS proteins also appear to have specificity in terms of which GPCRs they modulate, even when those GPCRs signal through the same type of G-proteins. The dopamine D2 receptor and 5-HT1A receptors are both Gα_i/o_ coupled GPCRs, however, RGS4, RGS10 and RGSZ1 reduced 5-HT1A receptor-mediated signaling *in vitro* but did not affect D2-mediated signaling (Ghavami et al., 2003). It remains unclear whether RGS proteins achieve this specificity through direct interaction with certain GPCRs or by interactions with other intracellular binding partners.

Membrane anchoring can also affect how RGS proteins regulate G-protein action. For example, palmitoylation of RGS16 is necessary for regulation of Gα_i_ and Gα_q_ signaling (Hiol et al., 2003). RGS proteins in complex with a Gα subunit can also directly affect signaling to downstream effectors, independent of their GAP function. This level of regulation also depends on the specific RGS protein involved, for instance RGS2 in complex with Gα_q_ can prevent Gα_q_ from binding to the downstream effectors p63 RhoGEF and GRK2, while RGS4 in complex with Gα_q_ has little effect on binding to these effectors (Shankaranarayanan et al., 2008).

Therefore, while different RGS proteins have classically been thought to have largely redundant actions, current evidence suggests that different family members might have considerable variation in G-protein preference, receptor selectivity, and scaffolding functions toward downstream effectors. This highlights the need for a better understanding of what role the RGS proteins play in neuropsychiatric disorders and treatments.

## RGS Insensitive G-Proteins

When an individual RGS protein is knocked out or genetically modified, other available RGS proteins can at least partially compensate. Similarly, when an individual G-protein is lost, a GPCR may be able to continue signaling through other available G-proteins. In order to overcome these difficulties a series of modified G-proteins were developed which are insensitive to the negative regulatory effects of RGS proteins. These mutated G proteins have a Gly in the “switch 3” region of the Gα protein that binds RGS proteins, replaced with a Ser (Tesmer et al., 1997). This prevents the protein-protein interaction necessary for GAP activity.

The potential utility of RGS insensitive (RGSi) G-proteins was quickly recognized and a series of novel mammalian RGSi G-proteins were created (DiBello et al., 1998; Lan et al., 1998). These mutations do not affect the kinetics of GDP release, GTP hydrolysis, Gβγ binding, or interaction with the receptor, but produce up to 100-fold loss of affinity for RGS proteins (Day et al., 2004; Fu et al., 2004). These properties allowed investigators to probe the effects of removing all RGS control at a specific G-protein without affecting G-protein signaling otherwise, avoiding the difficulties posed by RGS protein redundancy.

Mice expressing an RGSi knock-in variant of Gα_i2_ display a profound antidepressant-like phenotype across a number of behavioral tests including tail suspension, forced swim, elevated plus maze and novelty induced hypophagia (Talbot et al., 2010; Senese et al., 2018). These mice also have antidepressant-like signaling changes in the hippocampus and frontal cortex, including reduced glycogen synthase kinase 3 beta (GSK3β) activity (Talbot et al., 2010). GSK3β inhibition produces neurogenesis in the adult hippocampus, and this neurogenic effect may be an important component of antidepressant action (Malberg et al., 2000; Li et al., 2004; Tsai et al., 2008), although the significance of these antidepressant induced neurogenic effects has been disputed (Hanson et al., 2011).

Both the changes in hippocampal GSK3β and antidepressant-like behaviors observed in RGSi Gα_i2_ knock-in mice are fully reversed by pretreatment with a 5-HT1A receptor antagonist (Talbot et al., 2010). Furthermore, direct application of this 5-HT1A receptor antagonist to the hippocampus of the RGSi Gα_i2_ knock-in mice reversed their antidepressant-like behavior (Senese et al., 2018), suggesting that enhanced 5-HT1A receptor activity in the hippocampus is necessary for this behavior. Coupled with the fact that 5-HT1A receptor agonists produce more potent antidepressant-like effects in these animals, it appears that the loss of RGS control at Gα_i2_ promotes 5-HT1A receptor signaling leading to robust antidepressant-like effects. Mice heterozygous for RGSi Gα_o_ also show antidepressant-like effects in the tail suspension test, but the GPCR involved is not the 5-HT1A receptor and remains unidentified (Senese et al., 2018). The culprit in this case may be the delta opioid receptor (DOPR) since the potency of the delta agonist SNC80 to produce antidepressant-like effects is enhanced in these mice (Dripps et al., 2018).

Although activating postsynaptic 5-HT1A heteroreceptors is generally considered beneficial for antidepressant action, presynaptic 5-HT1A autoreceptor activation can limit antidepressant action and may contribute to the hysteresis observed between initiation of treatment and the onset of therapeutic effects (Hjorth and Sharp, 1993; Artigas et al., 1994; Le Poul et al., 1995; Matsuda et al., 1995). Interestingly, RGSi Gα_i2_ knock-in mice display enhancements of responses known to depend on 5-HT1A heteroreceptor activity (e.g., antidepressant-like behaviors, hippocampal GSK3β inhibition), but not responses dependent on 5-HT1A autoreceptor activity (e.g., hypothermia; Hillegaart, 1991; Matsuda et al., 1995; Li et al., 2004; Talbot et al., 2010). 5-HT1A receptor agonists also produce exaggerated responses in hippocampal neurons from these mice, with more robust 5-HT1A receptor dependent suppression of neuronal excitability observed in pyramidal neurons (Senese et al., 2018). As the hippocampus does not contain 5-HT cell bodies (Ren et al., 2018), this provides direct evidence for increased 5-HT1A heteroreceptor activity in these animals.

The above data suggest that disrupting RGS control of Gα_i2_ may represent a novel strategy to selectively enhance the antidepressant effects of 5-HT1A receptor activation without promoting the detrimental effects of autoreceptor activation. Unfortunately, specific RGS protein(s) responsible for the antidepressant-like phenotype in the RGSi-Gα_i2_ mice are not currently known, but two candidates (RGS6 and RGS19) are discussed later in this review.

Although the above studies highlight roles for RGS proteins in modulating depressive-like behaviors in preclinical models, they do not identify which RGS protein or proteins are involved. However, a limited number of studies have been performed on specific RGS proteins as described below.

## RGS2

Mice lacking RGS2 showed a baseline increase in anxious and depressive-like behaviors (Oliveira-dos-Santos et al., 2000; Lifschytz et al., 2012). These behavioral alterations occur alongside decreased presynaptic 5-HT1A receptor expression and function, suggesting that these receptors may play a role in the observed behavioral phenotype (Lifschytz et al., 2012; Muma, 2012 for review). In contrast, a genetic manipulation which specifically increases raphe 5-HT1A receptor expression in mice increases vulnerability to stress and decreases response to antidepressants (Richardson-Jones et al., 2010), suggesting that reductions in raphe 5-HT1A receptor availability following disruption of RGS2 may be a compensatory change rather than a causative factor of the pro-depressant behaviors. Nonetheless, these data demonstrate that RGS2 may have a protective effect against anxiety and depression, and that RGS2 disruption may have detrimental neuropsychiatric effects.

In line with these pre-clinical results, individuals expressing any of several single nucleotide polymorphisms (SNPs) in the RGS2 gene experience anxiety disorders and suicidal ideations at an increased rate (Leygraf et al., 2006; Smoller et al., 2008; Amstadter et al., 2009). In addition, various RGS2 genetic variants were found at an increased rate in suicide completers than controls (Cui et al., 2008). However, an increase in RGS2 immunoreactivity was also found in post-mortem tissue from both the prefrontal cortex and amygdala of these subjects (Cui et al., 2008), although this may represent a compensatory upregulation due to a loss of RGS2 functionality. Together these findings provide solid evidence that genetic alterations in the RGS2 gene can influence the development or expression of affective disorders in human populations, in line with findings from RGS2 knockout mice.

While it remains difficult to study antidepressant action *in vitro*, a number of reports have provided evidence on how RGS2 can affect cellular processes in ways that might modify antidepressant action. For example, an increase in hippocampal synaptic plasticity typically occurs following antidepressant treatment, while depressive states cause decreased plasticity (Kozisek et al., 2008; Nissen et al., 2010). Loss of RGS2 produces a similar loss of synaptic plasticity in mouse hippocampal tissue potentially by altering Gα_i/o_-mediated inhibition of hippocampal Ca^2+^ channels (Han et al., 2006). This suggests that a loss of neural plasticity due to genetic variation in RGS2 could have detrimental neuropsychiatric effects and might impair the function of antidepressant drugs.

## RGS4

Studies in rodent models have consistently shown that 5-HT1A receptor agonists such as 8-OH-DPAT cause a reduction in extracellular serotonin levels in the brain by activating 5-HT1A autoreceptors in the dorsal raphe nucleus (Casanovas and Artigas, 1996; Adell and Artigas, 1998; Celada et al., 2001). It is thought that this 5-HT1A receptor-dependent reduction in central serotonin may delay the beneficial effects of SSRI antidepressants. Indeed, strategies to limit 5-HT1A autoreceptor activity during SSRI treatment, such as co-administration of 5-HT1A receptor antagonists or weak partial agonists, have shown promising results (Artigas et al., 1994; Pérez et al., 1997; Tome et al., 1997; Maes et al., 1999). Although RGS4 mRNA is not normally expressed in the dorsal raphe nucleus (Gold et al., 1997), RGS4 overexpression in this region attenuates the ability of 5-HT1A receptors to reduce central serotonin levels (Beyer et al., 2004). Based on these results, overexpression or stimulation of RGS4 in brain regions containing 5-HT1A autoreceptors should have beneficial effects on antidepressant drug action, similar to the results obtained by combining 5-HT1A antagonists with traditional antidepressants (Artigas et al., 1994).

Delta opioid receptor agonists produce antidepressant-like behavioral effects in rodent models including the tail suspension and forced swim tests (Broom et al., 2002; Naidu et al., 2007). RGS4 knockout mice show an enhanced antidepressant-like response to DOPR agonists in the forced swim test, but not tail suspension test, suggesting that these antidepressant-like behaviors may depend on distinct neural pathways or signaling intermediates downstream of DOPR activation (Stratinaki et al., 2013; Dripps et al., 2017). This effect on forced swim test behavior appears to depend on nucleus accumbens RGS4 expression, as specific RGS4 knockdown in this region produces similar effects as global RGS4 knockout (Stratinaki et al., 2013). RGS proteins capable of modulating DOPR-mediated tail suspension test behavior have not yet been identified.

In addition to inhibiting the antidepressant-like effects of DOPR agonists, loss of RGS4 appears to inhibit the antidepressant-like effects of SSRIs, norepinephrine reuptake inhibitors and the *N*-methyl-D-aspartate (NMDA) receptor antagonist ketamine (Stratinaki et al., 2013). Acute treatment with either a DOPR agonist or ketamine decreases frontal cortex RGS4 expression yet only chronic treatment with a DOPR agonist increases RGS4 expression in the nucleus accumbens (Stratinaki et al., 2013). While this would suggest that nucleus accumbens and frontal cortex RGS4 have opposing effects on antidepressant action, this has yet to be conclusively demonstrated. Although RGS4 expression in the brain does not appear to differ between post-mortem tissue from depressed and healthy individuals, an upregulation of RGS4 has been observed in post-mortem nucleus accumbens tissue from depressed individuals undergoing antidepressant treatment compared to untreated depressed individuals (Stratinaki et al., 2013). Coupled with findings from rodent models showing increased antidepressant effectiveness in animals with overexpression of RGS4 in this brain region, it appears possible that nucleus accumbens RGS4 has a facilitatory effect on antidepressant treatment.

The RGS4 inhibitor CCG-203769 produces antidepressant-like effects on the tail suspension test in a mouse model (Senese et al., 2018). As RGS4 knockout mice do not have behavioral changes on the tail suspension test (Dripps et al., 2017), and loss of RGS4 inhibits the antidepressant-like effects of drugs with diverse mechanisms of action (Stratinaki et al., 2013), RGS4 inhibition alone cannot explain the antidepressant-like effect of CCG-203769. However, CCG-203769 also inhibits RGS19, and downregulation of RGS19 has been shown to increase 5-HT1A receptor signaling in hippocampal neurons (Wang et al., 2014). CCCG-203769’s antidepressant-like effects are discussed in more detail in the section on RGS19.

## RGS6

Mice lacking RGS6 display antidepressant-like and anxiolytic behaviors at baseline, including in the elevated plus maze and novelty induced hyponeophagia test (Stewart et al., 2014). This behavioral phenotype is fully reversible by 5-HT1A receptor antagonist pretreatment, and by direct activation of adenylyl cyclase with forskolin (Stewart et al., 2014). Loss of RGS6 did not affect mitogen-activated protein kinase (MAPK) or GSK3β signaling (Stewart et al., 2014), changes that have been detected in mice with a 5-HT1A receptor dependent antidepressant-like phenotype due to loss of RGS control at Gα_i2_ (Talbot et al., 2010). Instead the RGS6 knockout mouse phenotype appears to depend on increased phospho-CREB in the hippocampus and cortex, areas with high 5-HT1A receptor expression (Stewart et al., 2014). The mechanistic differences across the two mouse models calls into question whether RGS6 is the primary mediator of the RGSi-Gα_i2_ knock-in mouse behavioral phenotype, although it may be one of several RGS proteins involved. Nonetheless, the findings suggest that RGS6 may normally limit the actions of serotonergic antidepressants by reducing adenylyl cyclase inhibition downstream of 5-HT1A receptor activation, and that strategies to limit RGS6 activity may have beneficial effects for antidepressant treatment.

## RGS8

Overexpression of RGS8 in mouse brain produces an antidepressant-like behavioral phenotype in the forced swim test (Kobayashi et al., 2018). RGS8 is a potent negative modulator of melanin-concentrating hormone receptor 1 (MCHR1) signaling (Miyamoto-Matsubara et al., 2008). Inhibitors of MCHR1 have antidepressant-like effects, however, this action is lost in mice overexpressing RGS8, suggesting signaling downstream of MCHR1 is the target for RGS8. Whether loss of central RGS8 produces a pro-depressant phenotype has not yet been tested, although small molecule compounds with inhibitory action against RGS8 (Blazer et al., 2011; Storaska et al., 2013) could provide the means to examine these behaviors in future studies. While these RGS8 inhibitors are not highly potent or selective, a detailed analysis of the binding sites for these inhibitors (Shaw et al., 2018) will facilitate the discovery of more selective compounds.

## RGS16

Although RGS16 has not been directly linked to depression, palmitoylation causes the accumulation of RGS16 in lipid raft domains, a subcellular membrane compartment known to generally promote signaling downstream of Gα_q._ (Rybin et al., 2000; Hiol et al., 2003). As discussed earlier, this localization places RGS16 at or near the putative, but not yet positively identified, binding site for antidepressant drugs within lipid rafts (Eisensamer et al., 2005; Erb et al., 2016). This localization may provide RGS16 with an increased ability to regulate antidepressant drug action, although this prediction has yet to be tested.

## RGS17 (RGSZ2)

Levels of RGS17 mRNA (also known as RGSZ2) were shown to be markedly reduced in post-mortem brain tissue from individuals diagnosed with MDD (Shelton et al., 2011) in a large-scale RNA microarray analysis. Unfortunately, this study did not provide a potential mechanism by which MDD affects RGS17, nor evidence that these changes in RGS17 mRNA reflect changes in RGS17 activity.

## RGS19 (Gaip)

RGS19, also known as G Alpha Interacting Protein (GAIP; De Vries et al., 1995) has been shown to regulate 5-HT1A receptor signaling in both C6 and SH-SY5Y cells (Wang et al., 2014). RGS19 knockdown facilitated 5-HT1A receptor agonist induced activation of MAPK and inhibition of adenylyl cyclase (Wang et al., 2014), while RGS4 knockdown did not significantly affect signaling. The effect of RGS19 knockdown was magnified when the cells were co-treated with fibroblast growth factor 2 (FGF2), a factor known to act synergistically with 5-HT1A receptor activity in the hippocampus to facilitate synaptic plasticity (Borroto-Escuela et al., 2012; Wang et al., 2014). The enhancement of 5-HT1A receptor agonist stimulated MAPK activity following RGS19 knockdown seen in cellular models was replicated in mouse primary hippocampal neurons, including the synergistic enhancement by co-treatment with FGF2 and a 5-HT1A receptor agonist (Wang et al., 2014). This suggests that reducing RGS19 activity may facilitate the action of serotonergic antidepressants due to disinhibition of hippocampal 5-HT1A receptor activity.

CCG-203769, an RGS inhibitor with 100-fold selectivity for RGS19 and RGS4 compared to other RGS proteins (Blazer et al., 2015), produces antidepressant-like effects in a mouse model (Senese et al., 2018). These effects occur after repeated intra-hippocampal administration in female, but not male mice (Senese et al., 2018), suggesting that behavioral responses to RGS inhibitors may be sexually dimorphic. This behavioral sex difference occurs despite similar hippocampal expression of RGS19 between male and female mice (Senese et al., 2018).

As discussed above, loss of RGS4 activity attenuates the effects of various antidepressants (Stratinaki et al., 2013), suggesting that the antidepressant-like behavioral effects observed following CCG-203769 administration are not likely to be mediated by its inhibitory effect on RGS4 (Blazer et al., 2015; Senese et al., 2018). In contrast, loss of RGS19 activity facilitates 5-HT1A receptor mediated MAPK activity (Wang et al., 2014), signaling changes that have been associated with antidepressant-like behaviors in animal models (Talbot et al., 2010). While not conclusive, these data suggest that the antidepressant-like effects of CCG-203769 in female mice are due to its inhibitory effects on RGS19 (Senese et al., 2018). This is the first report of antidepressant-like behaviors produced by a compound which targets RGS proteins directly, although the requirement for central delivery of CCG-203769, and its action as an irreversible inhibitor, suggests that novel RGS inhibitors with improved drug-like properties are needed before this class of compounds can be fully explored for potential clinical utility.

## RGS20 (RGSZ1)

In post-mortem tissue from individuals with MDD, RGS20 (also known as RGSZ1) mRNA is significantly decreased in the anterior cingulate cortex (Tomita et al., 2013). This change was not observed in tissue from individuals with bipolar disorder (Tomita et al., 2013). It remains unclear whether this change in RGS20 mRNA is reflected by changes in protein expression or activity. Furthermore, as the majority of these individuals were undergoing neuropsychiatric treatment at time of death, it is impossible to determine whether RGS20 mRNA was reduced due to antidepressant treatment, or reflects an innate difference in the depressed brain.

Chronic estradiol treatments both desensitize hypothalamic 5-HT1A receptors and cause an increase in plasma membrane RGS20 expression (Carrasco et al., 2004; McAllister et al., 2014). Increased RGS20 is predicted to reduce signaling downstream of 5-HT1A receptor and therefore may contribute to the observed 5-HT1A receptor desensitization, although this interaction has not been conclusively demonstrated (Carrasco et al., 2004). 5-HT1A receptor desensitization in the dorsal raphe nucleus, not the hypothalamus, is generally considered a critical step in antidepressant action. However, RGS20 expression in the dorsal raphe has not been assessed following chronic antidepressant treatment, so it remains possible that a similar process contributes to 5-HT1A receptor desensitization in this brain region.

## Conclusion

G-proteins and their RGS protein modulators likely play important roles in the development of depressive states, and also influence the effectiveness of antidepressant therapies. Changes in activity of these proteins can have dramatic effects on these complex disorders, while even more subtle alterations, such as G-protein translocation between subcellular microdomains, can profoundly regulate antidepressant action. Although preclinical studies provide multiple hypotheses for how these proteins behave in depressed populations in the clinic, only a handful of these theories have been addressed in humans. Considering how alterations in G-proteins and/or RGS differentially affect responses to a variety of antidepressant treatments, it appears reasonable that a better understanding of these proteins could aid in the tailoring of personalized treatment strategies for depression. Screening for changes in G-protein signaling pathways could also provide new insight into susceptibility toward depressive disorders at an individual level. In addition, direct RGS-inhibiting compounds have been proposed as novel treatment options for a variety of indications, and selective small molecule RGS inhibitors have already been identified (Zhong and Neubig, 2001; Roman et al., 2006; Shaw et al., 2018). A more complete understanding of how these G-proteins and their partners interact with antidepressant therapies and the development of depressive states is both needed and welcome.

## Author Contributions

NS provided the original draft, and editing of subsequent drafts. MR and JT provided project funding, and manuscript review and editing.

## Conflict of Interest Statement

The authors declare that the research was conducted in the absence of any commercial or financial relationships that could be construed as a potential conflict of interest.

## References

[B1] Adell A. Artigas F. (1998). A microdialysis study of the *in vivo* release of 5-HT in the median raphe nucleus of the rat. *Br. J. Pharmacol.* 125 1361–1367. 10.1038/sj.bjp.0702206 9863668 PMC1565710

[B2] Allen J. A. Halverson-Tamboli R. A. Rasenick M. M. (2007). Lipid raft microdomains and neurotransmitter signalling. *Nat. Rev. Neurosci.* 8 128–140. 10.1038/nrn2059 17195035

[B3] Allen J. A. Yu J. Z. Dave R. H. Bhatnagar A. Roth B. L. Rasenick M. M. (2009). Caveolin-1 and lipid microdomains regulate gs trafficking and attenuate Gs/Adenylyl cyclase signaling. *Mol. Pharmacol.* 76 1082–1093. 10.1124/mol.109.060160 19696145 PMC2774991

[B4] Allen J. A. Yu J. Z. Donati R. J. Rasenick M. M. (2005). β-Adrenergic receptor stimulation promotes gα_s_ internalization through lipid rafts: a study in living cells. *Mol. Pharmacol.* 67 1493–1504. 10.1124/mol.104.008342 15703379

[B5] Amstadter A. B. Koenen K. C. Ruggiero K. J. Acierno R. Galea S. Kilpatrick D. G. (2009). Variation in RGS2 is associated with suicidal ideation in an epidemiological study of adults exposed to the 2004 Florida Hurricanes. *Arch. Suicide Res.* 13 349–357. 10.1080/13811110903266541 19813112 PMC2760049

[B6] Artigas F. Perez V. Alvarez E. (1994). Pindolol induces a rapid improvement of depressed patients treated with serotonin reuptake inhibitors. *Arch. Gen. Psychiatry* 51 248–251. 10.1001/archpsyc.1994.03950030084009 8122960

[B7] Beyer C. E. Ghavami A. Lin Q. Sung A. Rhodes K. J. Dawson L. A. (2004). Regulators of G-protein signaling 4: modulation of 5-HT1A-mediated neurotransmitter release in vivo. *Brain Res.* 1022 214–220. 10.1016/j.brainres.2004.06.073 15353231

[B8] Bhatnagar A. Sheffler D. J. Kroeze W. K. Compton-Toth B. Roth B. L. (2004). Caveolin-1 interacts with 5-HT2A serotonin receptors and profoundly modulates the signaling of selected Gαq -coupled protein receptors. *J. Biol. Chem.* 279 34614–34623. 10.1074/jbc.M404673200 15190056

[B9] Blazer L. L. Storaska A. J. Jutkiewicz E. M. Turner E. M. Calcagno M. Wade S. M. (2015). Selectivity and anti-parkinson’s potential of thiadiazolidinone RGS4 inhibitors. *ACS Chem. Neurosci.* 6 911–919. 10.1021/acschemneuro.5b00063 25844489

[B10] Blazer L. L. Zhang H. Casey E. M. Husbands S. M. Neubig R. R. (2011). A nanomolar-potency small molecule inhibitor of regulator of g-protein signaling proteins. *Biochemistry* 50 3181–3192. 10.1021/bi1019622 21329361 PMC3090679

[B11] Borroto-Escuela D. O. DuPont C. M. Li X. Savelli D. Lattanzi D. Srivastava I. (2017). Disturbances in the FGFR1-5-HT1A heteroreceptor complexes in the raphe-hippocampal 5-HT system develop in a genetic rat model of depression. *Front. Cell. Neurosci.* 11:309. 10.3389/fncel.2017.00309 29066953 PMC5641403

[B12] Borroto-Escuela D. O. Romero-Fernandez W. Mudó G. Pérez-Alea M. Ciruela F. Tarakanov A. O. (2012). Fibroblast growth factor receptor 1– 5-hydroxytryptamine 1A heteroreceptor complexes and their enhancement of hippocampal plasticity. *Biol. Psychiatry* 71 84–91. 10.1016/j.biopsych.2011.09.012 22035699

[B13] Broom D. Jutkiewicz E. M. Folk J. E. Traynor J. R. Rice K. C. Woods J. H. (2002). Nonpeptidic δ-opioid receptor agonists reduce immobility in the forced swim assay in rats. *Neuropsychopharmacology* 26 744–755. 10.1016/S0893-133X(01)00413-4 12007745

[B14] Carrasco G. Barker S. A. Zhang Y. Damjanoska K. J. Sullivan N. R. Garcia F. (2004). Estrogen treatment increases the levels of regulator of G-protein signaling-Z1 in the hypothalamic paraventricular nucleus: possible role in desensitization of 5-hydroxytryptamine1A receptors. *Neuroscience* 127 261–267. 10.1016/j.neuroscience.2004.05.031 15262317

[B15] Casanovas J. M. Artigas F. (1996). Differential effects of ipsapirone on 5-hydroxytryptamine release in the dorsal and median raphe neuronal pathways. *J. Neurochem.* 67 1945–1952. 10.1046/j.1471-4159.1996.67051945.x 8863499

[B16] Celada P. Puig M. V. Casanovas J. M. Guillazo G. Artigas F. (2001). Control of dorsal raphe serotonergic neurons by the medial prefrontal cortex: involvement of serotonin-1A, GABA(A), and glutamate receptors. *J. Neurosci.* 21 9917–9929. 10.1523/JNEUROSCI.21-24-09917.2001 11739599 PMC6763042

[B17] Chen J. Rasenick M. M. (1995a). Chronic antidepressant treatment facilitates G-protein activation of adenylyl cyclase without altering G-protein content. *J. Pharmacol. Exp. Ther.* 275 509–517. 7562593

[B18] Chen J. Rasenick M. M. (1995b). Chronic treatment of C6 glioma cells with antidepressant drugs increases functional coupling between a G-protein (Gs) and adenylyl cyclase. *J. Neurochem.* 64 724–732. 10.1046/j.1471-4159.1995.64020724.x 7830066

[B19] Cowburn R. F. Marcusson J. O. Eriksson A. Wiehager B. O’Neill C. (1994). Adenylyl cyclase activity and G-protein subunit levels in postmortem frontal cortex of suicide victims. *Brain Res.* 633 297–304. 10.1016/0006-8993(94)91552-0 8137164

[B20] Cui H. Nishiguchi N. Ivleva E. Yanagi M. Fukutake M. Nushida H. (2008). Association of RGS2 gene polymorphisms with suicide and increased RGS2 immunoreactivity in the postmortem brain of suicide victims. *Neuropsychopharmacology* 33 1537–1544. 10.1038/sj.npp.1301557 17728697

[B21] Czysz A. H. Schappi J. M. Rasenick M. M. (2015). Lateral diffusion of Gα_s_ in the plasma membrane is decreased after chronic but not acute antidepressant treatment: role of lipid raft and non-raft membrane microdomains. *Neuropsychopharmacology* 40 766–773. 10.1038/npp.2014.256 25249058 PMC4289966

[B22] Day P. W. Tesmer J. J. G. Sterne-Marr R. Freeman L. C. Benovic J. L. Wedegaertner P. B. (2004). Characterization of the GRK2 binding site of Galphaq. *J. Biol. Chem.* 279 53643–53652. 10.1074/jbc.M401438200 15471870 PMC1432089

[B23] De Montis G. M. Devoto P. Gessa G. L. Porcella A. Serra G. Tagliamonte A. (1990). Selective adenylate cyclase increase in the limbic area of long-term imipramine-treated rats. *Eur. J. Pharmacol.* 180 169–174. 10.1016/0014-2999(90)90605-6 2194824

[B24] De Vries L. Mousli M. Wurmser A. Farquhar M. G. (1995). GAIP, a protein that specifically interacts with the trimeric G protein G alpha i3, is a member of a protein family with a highly conserved core domain. *Proc. Natl. Acad. Sci. U.S.A.* 92 11916–11920. 10.1073/pnas.92.25.11916 8524874 PMC40514

[B25] DiBello P. R. Garrison T. R. Apanovitch D. M. Hoffman G. Shuey D. J. Mason K. (1998). Selective uncoupling of RGS action by a single point mutation in the G-protein alpha-subunit. *J. Biol. Chem.* 273 5780–5784. 10.1074/jbc.273.10.5780 9488712

[B26] Dikeos D. G. Papadimitriou G. N. Avramopoulos D. Karadima G. Daskalopoulou E. G. Souery D. (1999). Association between the dopamine D3 receptor gene locus (DRD3) and unipolar affective disorder. *Psychiatr. Genet.* 9 189–195. 10.1097/00041444-199912000-00005 10697826

[B27] Dmitrzak-Weglarz M. Rybakowski J. K. Slopien A. Czerski P. M. Leszczynska-Rodziewicz A. Kapelski P. (2006). Dopamine receptor D1 Gene –48A/G polymorphism is associated with bipolar illness but not with schizophrenia in a polish population. *Neuropsychobiology* 53 46–50. 10.1159/000090703 16397404

[B28] Donati R. J. Dwivedi Y. Roberts R. C. Conley R. R. Pandey G. N. Rasenick M. M. (2008). Postmortem brain Tof depressed suicides reveals increased Gs localization in lipid raft domains where it is less likely to activate adenylyl cyclase. *J. Neurosci.* 28 3042–3050. 10.1523/JNEUROSCI.5713-07.2008 18354007 PMC6670711

[B29] Donati R. J. Rasenick M. M. (2005). Chronic antidepressant treatment prevents accumulation of Gsα in cholesterol-rich, cytoskeletal-associated, plasma membrane domains (lipid rafts). *Neuropsychopharmacology* 30 1238–1245. 10.1038/sj.npp.1300697 15726116

[B30] Donati R. J. Schappi J. Czysz A. H. Jackson A. Rasenick M. M. (2015). Differential effects of antidepressants escitalopram versus lithium on Gs alpha membrane relocalization. *BMC Neurosci.* 16:40. 10.1186/s12868-015-0178-y 26162823 PMC4499192

[B31] Dripps I. J. Boyer B. T. Neubig R. R. Rice K. C. Traynor J. R. Jutkiewicz E. M. (2018). Role of signalling molecules in behaviours mediated by the δ opioid receptor agonist SNC80. *Br. J. Pharmacol.* 175 891–901. 10.1111/bph.14131 29278419 PMC5825297

[B32] Dripps I. J. Wang Q. Neubig R. R. Rice K. C. Traynor J. R. Jutkiewicz E. M. (2017). The role of regulator of G protein signaling 4 in delta-opioid receptor-mediated behaviors. *Psychopharmacology (Berl).* 234 29–39. 10.1007/s00213-016-4432-5 27624599 PMC5203942

[B33] Dunphy J. T. Greentree W. K. Linder M. E. (2001). Enrichment of G-protein palmitoyltransferase activity in low density membranes. *J. Biol. Chem.* 276 43300–43304. 10.1074/jbc.M104275200 11557754

[B34] Dwivedi Y. Pandey G. N. (1997). Effects of subchronic administration of antidepressants and anxiolytics on levels of the alpha subunits of G-proteins in the rat brain. *J. Neural. Transm.* 104 747–760. 10.1007/BF01291891 9444573

[B35] Dwivedi Y. Rizavi H. S. Conley R. R. Roberts R. C. Tamminga C. A. Pandey G. N. (2002). mRNA and protein expression of selective alpha subunits of G-proteins are abnormal in prefrontal cortex of suicide victims. *Neuropsychopharmacology* 27 499–517. 10.1016/S0893-133X(02)00335-4 12377388

[B36] Ebstein R. P. Lerer B. Shapira B. Shemesh Z. Moscovich D. G. Kindler S. (1988). Cyclic AMP second-messenger signal amplification in depression. *Br. J. Psychiatry* 152 665–669. 10.1192/bjp.152.5.665 2844354

[B37] Eisensamer B. Uhr M. Meyr S. Gimpl G. Deiml T. Rammes G. (2005). Antidepressants and antipsychotic drugs colocalize with 5-HT3 receptors in raft-like domains. *J. Neurosci.* 25 10198–10206. 10.1523/JNEUROSCI.2460-05.2005 16267227 PMC6725799

[B38] Emamghoreishi M. Warsh J. J. Sibony D. Li P. P. (1996). Lack of effect of chronic antidepressant treatment on Gs and Gi alpha-subunit protein and mRNA levels in the rat cerebral cortex. *Neuropsychopharmacology* 15 281–287. 10.1016/0893-133X(95)00211-U 8873111

[B39] Erb S. J. Schappi J. M. Rasenick M. M. (2016). Antidepressants accumulate in lipid rafts independent of monoamine transporters to modulate redistribution of the G protein. *Gα_s_. J. Biol. Chem.* 291 19725–19733. 10.1074/jbc.M116.727263 27432886 PMC5025664

[B40] Farran B. (2017). An update on the physiological and therapeutic relevance of GPCR oligomers. *Pharmacol. Res.* 117 303–327. 10.1016/j.phrs.2017.01.008 28087443

[B41] Fu Y. Zhong H. Nanamori M. Mortensen R. M. Huang X. Lan K. (2004). RGS-Insensitive G-protein mutations to study the role of endogenous RGS proteins. *In Methods Enzymol.* 389 229–243. 10.1016/S0076-6879(04)89014-1 15313569

[B42] Fujita M. Richards E. M. Niciu M. J. Ionescu D. F. Zoghbi S. S. Hong J. (2017). cAMP signaling in brain is decreased in unmedicated depressed patients and increased by treatment with a selective serotonin reuptake inhibitor. *Mol. Psychiatry* 22 754–759. 10.1038/mp.2016.171 27725657 PMC5388600

[B43] García-Velázquez R. Jokela M. Rosenström T. H. (2017). Symptom severity and disability in psychiatric disorders: the U.S. Collaborative psychiatric epidemiology survey. *J. Affect. Disord.* 222 204–210. 10.1016/j.jad.2017.07.015 28711797

[B44] Ghavami A. Hunt R. A. Olsen M. A. Zhang J. Smith D. L. Kalgaonkar S. (2003). Differential effects of regulator of G-protein signaling (RGS) proteins on serotonin 5-HT1A, 5-HT2A, and dopamine D2 receptor-mediated signaling and adenylyl cyclase activity. *Cell. Signal.* 16 711–721. 10.1016/j.cellsig.2003.11.006 15093612

[B45] Gold S. J. Ni Y. G. Dohlman H. G. Nestler E. J. (1997). Regulators of G-protein signaling (RGS) proteins: region-specific expression of nine subtypes in rat brain. *J. Neurosci.* 17 8024–8037. 10.1523/JNEUROSCI.17-20-08024.1997 9315921 PMC6793903

[B46] Ha C. M. Park D. Kim Y. Na M. Panda S. Won S. (2015). SNX14 is a bifunctional negative regulator for neuronal 5-HT6 receptor signaling. *J. Cell Sci.* 128 1848–1861. 10.1242/jcs.169581 25795301 PMC6518326

[B47] Han J. Mark M. D. Li X. Xie M. Waka S. Rettig J. (2006). RGS2 Determines short-term synaptic plasticity in hippocampal neurons by regulating Gi/o- mediated inhibition of presynaptic Ca2 + channels. *Neuron* 51 575–586. 10.1016/j.neuron.2006.07.012 16950156

[B48] Hanson N. D. Owens M. J. Nemeroff C. B. (2011). Depression, antidepressants and neurogenesis: a critical reappraisal. *Neuropsychopharmacology* 36 2589–2602. 10.1038/npp.2011.220 21937982 PMC3230505

[B49] Head B. P. Patel H. H. Roth D. M. Murray F. Swaney J. S. Niesman I. R. (2006). Microtubules and actin microfilaments regulate lipid raft/caveolae localization of adenylyl cyclase signaling components. *J. Biol. Chem.* 281 26391–26399. 10.1074/jbc.M602577200 16818493

[B50] Heximer S. P. Watson N. Linder M. E. Blumer K. J. Hepler J. R. (1997). RGS2/G0S8 is a selective inhibitor of Gqalpha function. *Proc. Natl. Acad. Sci. U.S.A.* 94 14389–14393. 10.1073/pnas.94.26.14389 9405622 PMC24991

[B51] Hillegaart V. (1991). Effects of local application of 5-HT and 8-OH-DPAT into the dorsal and median raphe nuclei on core temperature in the rat. *Psychopharmacology (Berl).* 103 291–296. 10.1007/BF02244281 1829235

[B52] Hines L. M. Tabakoff B. WHO/ISBRA Study on State and Trait Markers of Alcohol Use and Dependence Investigators (2005). Platelet adenylyl cyclase activity: a biological marker for major depression and recent drug use. *Biol. Psychiatry* 58 955–962. 10.1016/j.biopsych.2005.05.040 16095566

[B53] Hiol A. Davey P. C. Osterhout J. L. Waheed A. A. Fischer E. R. Chen C. K. (2003). Palmitoylation regulates regulators of G-protein signaling (RGS) 16 function. *J. Biol. Chem.* 278 19301–19308. 10.1074/jbc.M210123200 12642593

[B54] Hjorth S. Sharp T. (1993). In vivo microdialysis evidence for central serotonin1A and serotonin1B autoreceptor blocking properties of the beta adrenoceptor antagonist (-)penbutolol. *J. Pharmacol. Exp. Therap.* 265 707–712. 8098761

[B55] Hoffmann R. Wilkinson I. R. McCallum J. F. Engels P. Houslay M. D. (1998). cAMP-specific phosphodiesterase HSPDE4D3 mutants which mimic activation and changes in rolipram inhibition triggered by protein kinase A phosphorylation of Ser-54: generation of a molecular model. *Biochem. J.* 333( Pt 1), 139–149. 10.1042/bj3330139 9639573 PMC1219566

[B56] Hollinger S. Hepler J. R. (2002). Cellular regulation of RGS proteins: modulators and integrators of G protein signaling. *Pharmacol. Rev.* 54 527–559. 10.1124/pr.54.3.527 12223533

[B57] Houslay M. D. Sullivan M. Bolger G. B. (1998). The multienzyme PDE4 cyclic adenosine monophosphate-specific phosphodiesterase family: intracellular targeting, regulation, and selective inhibition by compounds exerting anti-inflammatory and antidepressant actions. *Adv. Pharmacol.* 44 225–342. 10.1016/S1054-3589(08)60128-3 9547887

[B58] Insel T. R. Wang P. S. (2009). The STAR^∗^D trial: revealing the need for better treatments. *Psychiatr. Serv.* 60 1466–1467. 10.1176/ps.2009.60.11.1466 19880463

[B59] Kantor E. D. Rehm C. D. Haas J. S. Chan A. T. Giovannucci E. L. (2015). Trends in prescription drug use among adults in the United States from 1999-2012. *JAMA* 314:1818. 10.1001/jama.2015.13766 26529160 PMC4752169

[B60] Kessler R. C. Berglund P. Demler O. Jin R. Koretz D. Merikangas K. R. (2003). The epidemiology of major depressive disorder. *JAMA* 289:3095. 10.1001/jama.289.23.3095 12813115

[B61] Kobayashi Y. Takemoto R. Yamato S. Okada T. Iijima M. Uematsu Y. (2018). Depression-resistant phenotype in mice overexpressing regulator of G protein signaling 8 (RGS8). *Neuroscience* 383 160–169. 10.1016/j.neuroscience.2018.05.005 29758252

[B62] Kozisek M. E. Middlemas D. Bylund D. B. (2008). Brain-derived neurotrophic factor and its receptor tropomyosin-related kinase B in the mechanism of action of antidepressant therapies. *Pharmacol. Therapeut.* 117 30–51. 10.1016/j.pharmthera.2007.07.001 17949819

[B63] Krumins A. M. Gilman A. G. (2006). Targeted knockdown of G protein subunits selectively prevents receptor-mediated modulation of effectors and reveals complex changes in non-targeted signaling proteins. *J. Biol. Chem.* 281 10250–10262. 10.1074/jbc.M511551200 16446365

[B64] Lan K. L. Sarvazyan N. A. Taussig R. Mackenzie R. G. DiBello P. R. Dohlman H. G. (1998). A point mutation in Galphao and Galphai1 blocks interaction with regulator of G-protein signaling proteins. *J. Biol. Chem.* 273 12794–12797. 10.1074/jbc.273.21.12794 9582306

[B65] Lasoń W. Przewłocki R. (1993). The effect of chronic treatment with imipramine on the G proteins mRNA level in the rat hippocampus–an interaction with a calcium channel antagonist. *Pol. J. Pharmacol.* 45 219–226. 8401777

[B66] Le Poul E. Laaris N. Doucet E. Laporte A. M. Hamon M. Lanfumey L. (1995). Early desensitization of somato-dendritic 5-HT1A autoreceptors in rats treated with fluoxetine or paroxetine. *Naunyn-Schmiedeberg’s Arch. Pharmacol.* 352 141–148. 10.1007/BF00176767 7477436

[B67] Lerer B. Macciardi F. Segman R. H. Adolfsson R. Blackwood D. Blairy S. (2001). Variability of 5-HT2C receptor cys23ser polymorphism among European populations and vulnerability to affective disorder. *Mol. Psychiatry* 6 579–585. 10.1038/sj.mp.4000883 11526472

[B68] Lesch K. P. Aulakh C. S. Tolliver T. J. Hill J. L. Murphy D. L. (1991). Regulation of G-proteins by chronic antidepressant drug treatment in rat brain: tricyclics but not clorgyline increase Go alpha subunits. *Eur. J. Pharmacol.* 207 361–364. 10.1016/0922-4106(91)90012-7 1783004

[B69] Leygraf A. Hohoff C. Freitag C. Willis-Owen S. A. G. Krakowitzky P. Fritze J. (2006). Rgs 2 gene polymorphisms as modulators of anxiety in humans? *J. Neural Transm.* 113 1921–1925. 10.1007/s00702-006-0484-8 16736243

[B70] Li X. Zhu W. Roh M.-S. Friedman A. B. Rosborough K. Jope R. S. (2004). In vivo regulation of glycogen synthase kinase-3β (GSK3β) by serotonergic activity in mouse brain. *Neuropsychopharmacology* 29 1426–1431. 10.1038/sj.npp.1300439 15039769 PMC1986663

[B71] Lifschytz T. Broner E. C. Zozulinsky P. Slonimsky A. Eitan R. Greenbaum L. (2012). Relationship between Rgs2 gene expression level and anxiety and depression-like behaviour in a mutant mouse model: serotonergic involvement. *Int. J. Neuropsychopharmacol.* 15 1307–1318. 10.1017/S1461145711001453 22040681

[B72] López J. F. Chalmers D. T. Little K. Y. Watson S. J. (1998). A.E. bennett research award. regulation of serotonin1A, glucocorticoid, and mineralocorticoid receptor in rat and human hippocampus: implications for the neurobiology of depression. *Biol. Psychiatry* 43 547–573. 10.1016/S0006-3223(97)00484-8 9564441

[B73] López León S. Croes E. A. Sayed-Tabatabaei F. A. Claes S. Broeckhoven C. van Duijn C. M. (2005). The dopamine D4 receptor gene 48-base-pair-repeat polymorphism and mood disorders: a meta-analysis. *Biol. Psychiatry* 57 999–1003. 10.1016/j.biopsych.2005.01.030 15860340

[B74] Maes M. Libbrecht I. van Hunsel F. Campens D. Meltzer H. Y. (1999). Pindolol and mianserin augment the antidepressant activity of fluoxetine in hospitalized major depressed patients, including those with treatment resistance. *J. Clin. Psychopharmacol.* 19 177–182. 10.1097/00004714-199904000-00014 10211920

[B75] Malberg J. E. Eisch A. J. Nestler E. J. Duman R. S. (2000). Chronic antidepressant treatment increases neurogenesis in adult rat hippocampus. *J. Neurosci.* 20 9104–9110. 10.1523/JNEUROSCI.20-24-09104.2000 11124987 PMC6773038

[B76] Mansbach R. S. Brooks E. N. Chen Y. L. (1997). Antidepressant-like effects of CP-154,526, a selective CRF1 receptor antagonist. *Eur. J. Pharmacol.* 323 21–26. 10.1016/S0014-2999(97)00025-3 9105872

[B77] Massat I. Souery D. Del-Favero J. Van Gestel S. Serretti A. Macciardi F. (2002). Positive association of dopamine D2 receptor polymorphism with bipolar affective disorder in a European Multicenter Association Study of affective disorders. *Am. J. Med. Genet.* 114 177–185. 10.1002/ajmg.10118 11857579

[B78] Matsuda T. Somboonthum P. Suzuki M. Asano S. Baba A. (1995). Antidepressant-like effect by postsynaptic 5-HT1A receptor activation in mice. *Eur. J. Pharmacol.* 280 235–238. 10.1016/0014-2999(95)00254-I 7589193

[B79] Matussek N. Ackenheil M. Hippius H. Müller F. Schröder H. T. Schultes H. (1980). Effect of clonidine on growth hormone release in psychiatric patients and controls. *Psychiatry Res.* 2 25–36. 10.1016/0165-1781(80)90004-9 6251501

[B80] McAllister C. E. Mi Z. Mure M. Li Q. Muma N. A. (2014). GPER1 stimulation alters posttranslational modification of rgsz1 and induces desensitization of 5-HT 1A receptor signaling in the rat hypothalamus. *Neuroendocrinology* 100 228–239. 10.1159/000369467 25402859 PMC4305009

[B81] Menkes D. B. Rasenick M. M. Wheeler M. A. Bitensky M. W. (1983). Guanosine triphosphate activation of brain adenylate cyclase: enhancement by long-term antidepressant treatment. *Science* 219 65–67. 10.1126/science.6849117 6849117

[B82] Miura Y. Hanada K. Jones T. L. (2001). G(s) signaling is intact after disruption of lipid rafts. *Biochemistry* 40 15418–15423. 10.1021/bi015574a 11735426

[B83] Miyamoto-Matsubara M. Saitoh O. Maruyama K. Aizaki Y. Saito Y. (2008). Regulation of melanin-concentrating hormone receptor 1 signaling by RGS8 with the receptor third intracellular loop. *Cell. Signal.* 20 2084–2094. 10.1016/j.cellsig.2008.07.019 18760349

[B84] Mooney J. J. Samson J. A. McHale N. L. Pappalarado K. M. Alpert J. E. Schildkraut J. J. (2013). Increased Gsα within blood cell membrane lipid microdomains in some depressive disorders: an exploratory study. *J. Psychiatr. Res.* 47 706–711. 10.1016/j.jpsychires.2013.02.005 23490066 PMC3669544

[B85] Muma N. A. (2012). RGS proteins: impact on the treatment of depression and anxiety. *Int. J. Neuropsychopharmacol.* 15 1199–1200. 10.1017/S1461145711002008 22277123

[B86] Naidu P. S. Lichtman A. H. Archer C. C. May E. L. Harris L. S. Aceto M. D. (2007). NIH 11082 produces anti-depressant-like activity in the mouse tail-suspension test through a delta-opioid receptor mechanism of action. *Eur. J. Pharmacol.* 566 132–136. 10.1016/j.ejphar.2007.03.031 17459369 PMC1939727

[B87] Naumenko V. S. Popova N. K. Lacivita E. Leopoldo M. Ponimaskin E. G. (2014). Interplay between serotonin 5-HT 1A and 5-HT 7 receptors in depressive disorders. *CNS Neurosci. Ther.* 20 582–590. 10.1111/cns.12247 24935787 PMC6493079

[B88] Nissen C. Holz J. Blechert J. Feige B. Riemann D. Voderholzer U. (2010). Learning as a model for neural plasticity in major depression. *Biol. Psychiatry* 68 544–552. 10.1016/j.biopsych.2010.05.026 20655508

[B89] Oldham W. M. Hamm H. E. (2008). Heterotrimeric G-protein activation by G-protein-coupled receptors. *Nat. Rev. Mol. Cell Biol.* 9 60–71. 10.1038/nrm2299 18043707

[B90] Oliveira-dos-Santos A. J. Matsumoto G. Snow B. E. Bai D. Houston F. P. Whishaw I. Q. (2000). Regulation of T cell activation, anxiety, and male aggression by RGS2. *Proc. Natl. Acad. Sci. U.S.A.* 97 12272–12277. 10.1073/pnas.220414397 11027316 PMC17331

[B91] Ostrom R. S. Post S. R. Insel P. A. (2000). Stoichiometry and compartmentation in G protein-coupled receptor signaling: implications for therapeutic interventions involving G(s). *J. Pharmacol. Exp. Ther.* 294 407–412. 10900212

[B92] Ozawa H. Rasenick M. M. (1989). Coupling of the stimulatory GTP-binding protein Gs to rat synaptic membrane adenylate cyclase is enhanced subsequent to chronic antidepressant treatment. *Mol. Pharmacol.* 36 803–808. 2511428

[B93] Ozawa H. Rasenick M. M. (1991). Chronic electroconvulsive treatment augments coupling of the GTP-binding protein Gs to the catalytic moiety of adenylyl cyclase in a manner similar to that seen with chronic antidepressant drugs. *J. Neurochem.* 56 330–338. 10.1111/j.1471-4159.1991.tb02599.x 1898967

[B94] Pandey G. N. Dwivedi Y. Ren X. Rizavi H. S. Mondal A. C. Shukla P. K. (2005). Brain region specific alterations in the protein and mRNA levels of protein kinase A subunits in the post-mortem brain of teenage suicide victims. *Neuropsychopharmacology* 30 1548–1556. 10.1038/sj.npp.1300765 15920506

[B95] Pandey G. N. Janicak P. Davis J. M. (1985). Studies of beta-adrenergic receptors in leukocytes of patients with affective illness and effects of antidepressant drugs. *Psychopharmacol. Bull.* 21 603–609. 2994162

[B96] Perez J. Tinelli D. Brunello N. Racagni G. (1989). cAMP-dependent phosphorylation of soluble and crude microtubule fractions of rat cerebral cortex after prolonged desmethylimipramine treatment. *Eur. J. Pharmacol.* 172 305–316. 10.1016/0922-4106(89)90060-6 2550266

[B97] Pérez V. Gilaberte I. Faries D. Alvarez E. Artigas F. (1997). Randomised, double-blind, placebo-controlled trial of pindolol in combination with fluoxetine antidepressant treatment. *Lancet* 349 1594–1597. 10.1016/S0140-6736(96)08007-5 9174562

[B98] Pesanová Z. Novotnı J. Cernı J. Milligan G. Svoboda P. (1999). Thyrotropin-releasing hormone-induced depletion of G(q)alpha/G(11)alpha proteins from detergent-insensitive membrane domains. *FEBS Lett.* 464 35–40. 10.1016/S0014-5793(99)01666-X 10611479

[B99] Post R. M. Ketter T. A. Joffe R. T. Kramlinger K. L. (1991). Lack of beneficial effects of l-baclofen in affective disorder. *Int. Clin. Psychopharmacol.* 6 197–207. 10.1097/00004850-199100640-00001 1816278

[B100] Ren J. Friedmann D. Xiong J. Liu C. D. Ferguson B. R. Weerakkody T. (2018). Anatomically defined and functionally distinct dorsal raphe serotonin sub-systems. *Cell* 175 472.e20–487.e20. 10.1016/j.cell.2018.07.043 30146164 PMC6173627

[B101] Renner U. Zeug A. Woehler A. Niebert M. Dityatev A. Dityateva G. (2012). Heterodimerization of serotonin receptors 5-HT1A and 5-HT7 differentially regulates receptor signalling and trafficking. *J. Cell Sci.* 125 2486–2499. 10.1242/jcs.101337 22357950

[B102] Richardson-Jones J. W. Craige C. P. Guiard B. P. Stephen A. Metzger K. L. Kung H. F. (2010). 5-HT1A autoreceptor levels determine vulnerability to stress and response to antidepressants. *Neuron* 65 40–52. 10.1016/j.neuron.2009.12.003 20152112 PMC2941196

[B103] Rivera-Baltanas T. Olivares J. M. Martinez-Villamarin J. R. Fenton E. Y. Kalynchuk L. E. Caruncho H. J. (2014). Serotonin 2A receptor clustering in peripheral lymphocytes is altered in major depression and may be a biomarker of therapeutic efficacy. *J. Affect. Disord.* 163 47–55. 10.1016/j.jad.2014.03.011 24836087

[B104] Roman D. L. Talbot J. N. Roof R. A. Sunahara R. K. Traynor J. R. Neubig R. R. (2006). Identification of small-molecule inhibitors of RGS4 using a high-throughput flow cytometry protein interaction assay. *Mol. Pharmacol.* 71 169–175. 10.1124/mol.106.028670 17012620

[B105] Rybin V. O. Xu X. Lisanti M. P. Steinberg S. F. (2000). Differential targeting of β-adrenergic receptor subtypes and adenylyl cyclase to cardiomyocyte caveolae. *J. Biol. Chem.* 275 41447–41457. 10.1074/jbc.M006951200 11006286

[B106] Senese N. B. Oginsky M. Neubig R. R. Ferrario C. Jutkiewicz E. M. Traynor J. R. (2018). Role of hippocampal 5-HT1A receptors in the antidepressant-like phenotype of mice expressing RGS-insensitive Gαi2 protein. *Neuropharmacology* 141 296–304. 10.1016/j.neuropharm.2018.09.002 30189184 PMC6392198

[B107] Shankaranarayanan A. Thal D. M. Tesmer V. M. Roman D. L. Neubig R. R. Kozasa T. (2008). Assembly of high order Gα q -effector complexes with RGS proteins. *J. Biol. Chem.* 283 34923–34934. 10.1074/jbc.M805860200 18936096 PMC2596395

[B108] Shaw V. S. Mohammadiarani H. Vashisth H. Neubig R. R. (2018). Differential protein dynamics of regulators of G-Protein signaling: role in specificity of small-molecule inhibitors. *J. Am. Chem. Soc.* 140 3454–3460. 10.1021/jacs.7b13778 29460621 PMC6309336

[B109] Shelton R. C. Claiborne J. Sidoryk-Wegrzynowicz M. Reddy R. Aschner M. Lewis D. A. (2011). Altered expression of genes involved in inflammation and apoptosis in frontal cortex in major depression. *Mol. Psychiatry* 16 751–762. 10.1038/mp.2010.52 20479761 PMC2928407

[B110] Simons K. Toomre D. (2000). Lipid rafts and signal transduction. *Nat. Rev. Mol. Cell Biol.* 1 31–39. 10.1038/35036052 11413487

[B111] Singh H. Wray N. Schappi J. M. Rasenick M. M. (2018). Disruption of lipid-raft localized Gα_s_/tubulin complexes by antidepressants: a unique feature of HDAC6 inhibitors, SSRI and tricyclic compounds. *Neuropsychopharmacology* 43 1481–1491. 10.1038/s41386-018-0016-x 29463911 PMC5983546

[B112] Smoller J. W. Paulus M. P. Fagerness J. A. Purcell S. Yamaki L. H. Hirshfeld-Becker D. (2008). Influence of RGS2 on anxiety-related temperament, personality, and brain function. *Arch. Gen. Psychiatry* 65:298. 10.1001/archgenpsychiatry.2007.48 18316676

[B113] Stewart A. Maity B. Wunsch A. M. Meng F. Wu Q. Wemmie J. A. (2014). Regulator of G-protein signaling 6 (RGS6) promotes anxiety and depression by attenuating serotonin-mediated activation of the 5-HT(1A) receptor-adenylyl cyclase axis. *FASEB J.* 28 1735–1744. 10.1096/fj.13-235648 24421401 PMC3963013

[B114] Stockmeier C. (2003). Involvement of serotonin in depression: evidence from postmortem and imaging studies of serotonin receptors and the serotonin transporter. *J. Psychiatr. Res.* 37 357–373. 10.1016/S0022-3956(03)00050-5 12849929

[B115] Storaska A. J. Mei J. P. Wu M. Li M. Wade S. M. Blazer L. L. (2013). Reversible inhibitors of regulators of G-protein signaling identified in a high-throughput cell-based calcium signaling assay. *Cell. Signal.* 25 2848–2855. 10.1016/j.cellsig.2013.09.007 24041654 PMC3848259

[B116] Stratinaki M. Varidaki A. Mitsi V. Ghose S. Magida J. Dias C. (2013). Regulator of G-protein signaling 4 is a crucial modulator of antidepressant drug action in depression and neuropathic pain models. *Proc. Natl. Acad. Sci. U.S.A.* 110 8254–8259. 10.1073/pnas.1214696110 23630294 PMC3657820

[B117] Svenningsson P. Chergui K. Rachleff I. Flajolet M. Zhang X. El Yacoubi M. (2006). Alterations in 5-HT1B receptor function by p11 in depression-like states. *Science* 311 77–80. 10.1126/science.1117571 16400147

[B118] Szafran K. Faron-Górecka A. Kolasa M. Kuśmider M. Solich J. Zurawek D. (2013). Potential role of G protein-coupled receptor (GPCR) heterodimerization in neuropsychiatric disorders: a focus on depression. *Pharmacol. Rep.* 65 1498–1505. 10.1016/S1734-1140(13)71510-X 24552997

[B119] Talbot J. N. Jutkiewicz E. M. Graves S. M. Clemans C. F. Nicol M. R. Mortensen R. M. (2010). RGS inhibition at G(alpha)i2 selectively potentiates 5-HT1A-mediated antidepressant effects. *Proc. Natl. Acad. Sci. U.S.A.* 107 11086–11091. 10.1073/pnas.1000003107 20534514 PMC2890727

[B120] Talbot J. N. Roman D. L. Clark M. J. Roof R. A. Tesmer J. J. G. Neubig R. R. (2009). Differential modulation of mu-opioid receptor signaling to adenylyl cyclase by regulators of G-protein signaling proteins 4 or 8 and 7 in permeabilised C6 cells is Gα subtype dependent. *J. Neurochem.* 112 1026–1034. 10.1111/j.1471-4159.2009.06519.x 20002516 PMC2947325

[B121] Tena-Campos M. Ramon E. Borroto-Escuela D. O. Fuxe K. Garriga P. (2015). The zinc binding receptor GPR39 interacts with 5-HT1A and GalR1 to form dynamic heteroreceptor complexes with signaling diversity. *Biochim. Biophys. Acta* 1852 2585–2592. 10.1016/j.bbadis.2015.09.003 26365466

[B122] Tesmer J. J. Berman D. M. Gilman A. G. Sprang S. R. (1997). Structure of RGS4 bound to AlF4–activated G(i alpha1): stabilization of the transition state for GTP hydrolysis. *Cell* 89 251–261. 10.1016/S0092-8674(00)80204-4 9108480

[B123] Toki S. Donati R. J. Rasenick M. M. (1999). Treatment of C6 glioma cells and rats with antidepressant drugs increases the detergent extraction of G(s alpha) from plasma membrane. *J. Neurochem.* 73 1114–1120. 10.1046/j.1471-4159.1999.0731114.x 10461902

[B124] Tome M. B. Isaac M. T. Harte R. Holland C. (1997). Paroxetine and pindolol: a randomized trial of serotonergic autoreceptor blockade in the reduction of antidepressant latency. *Int. Clin. Psychopharmacol.* 12 81–89. 10.1097/00004850-199703000-00003 9219043

[B125] Tomita H. Ziegler M. E. Kim H. B. Evans S. J. Choudary P. V. Li J. Z. (2013). G protein-linked signaling pathways in bipolar and major depressive disorders. *Front. Genet.* 4:297. 10.3389/fgene.2013.00297 24391664 PMC3870297

[B126] Traynor J. R. Neubig R. R. (2005). Regulators of G protein signaling & drugs of abuse. *Mol. Interv.* 5 30–41. 10.1124/mi.5.1.7 15734717

[B127] Tsai S. J. Liou Y. J. Hong C. J. Yu Y. W. Y. Chen T. J. (2008). Glycogen synthase kinase-3beta gene is associated with antidepressant treatment response in Chinese major depressive disorder. *Pharmacogenomics J.* 8 384–390. 10.1038/sj.tpj.6500486 18195729

[B128] Wang Q. Terauchi A. Yee C. H. Umemori H. Traynor J. R. (2014). 5-HT1A receptor-mediated phosphorylation of extracellular signal-regulated kinases (ERK1/2) is modulated by regulator of G-protein signaling protein 19. *Cell. Signal.* 26 1846–1852. 10.1016/j.cellsig.2014.04.017 24793302 PMC8019269

[B129] Wray N. H. Schappi J. M. Singh H. Senese N. B. Rasenick M. M. (2018). NMDAR-independent, cAMP-dependent antidepressant actions of ketamine. *Mol. Psychiatry* [Epub ahead of print]. 10.1038/s41380-018-0083-8 29895894 PMC8011999

[B130] Zhang L. Rasenick M. M. (2010). Chronic treatment with escitalopram but not R-citalopram translocates Galpha(s) from lipid raft domains and potentiates adenylyl cyclase: a 5-hydroxytryptamine transporter-independent action of this antidepressant compound. *J. Pharmacol. Exp. Ther.* 332 977–984. 10.1124/jpet.109.162644 19996298 PMC2835448

[B131] Zheng B. Ma Y. C. Ostrom R. S. Lavoie C. Gill G. N. Insel P. A. (2001). RGS-PX1, a GAP for galpha s and sorting nexin in vesicular trafficking. *Science* 294 1939–1942. 10.1126/science.1064757 11729322

[B132] Zhong H. Neubig R. R. (2001). Regulator of G-protein signaling proteins: novel multifunctional drug targets. *J. Pharmacol. Exp. Ther.* 297 837–845. 11356902

[B133] Zobel A. W. Nickel T. Künzel H. E. Ackl N. Sonntag A. Ising M. (2000). Effects of the high-affinity corticotropin-releasing hormone receptor 1 antagonist R121919 in major depression: the first 20 patients treated. *J. Psychiatr. Res.* 34 171–181. 10.1016/S0022-3956(00)00016-9 10867111

